# Laparoscopic Revisional Surgery After Failed Heller Myotomy for Esophageal Achalasia: Long-Term Outcome at a Single Tertiary Center

**DOI:** 10.1007/s11605-021-05041-x

**Published:** 2021-06-07

**Authors:** Giovanni Capovilla, Renato Salvador, Luca Provenzano, Michele Valmasoni, Lucia Moletta, Elisa Sefora Pierobon, Stefano Merigliano, Mario Costantini

**Affiliations:** Clinica Chirurgica 3, Department of Surgical, Oncological and Gastroenterological Sciences, Università di Padova, Via Giustiniani, 2, 35128 Padova, Italy

**Keywords:** Achalasia, Myotomy, Revisional surgery, Laparoscopy, Esophagectomy

## Abstract

**Background:**

Laparoscopic Heller myotomy (HM) has gained acceptance as the gold standard of treatment for achalasia. However, 10–20% of the patients will experience symptom recurrence, thus requiring further treatment including pneumodilations (PD) or revisional surgery. The aim of our study was to assess the long-term outcome of laparoscopic redo HM.

**Methods:**

Patients who underwent redo HM at our center between 2000 and 2019 were enrolled. Postoperative outcomes of redo HM patients (redo group) were compared with that of patients who underwent primary laparoscopic HM in the same time span (control group). For the control group, we randomly selected patients matched for age, sex, FU time, Eckardt score (ES), previous PD, and radiological stage. Failure was defined as an Eckardt score > 3 or the need for re-treatment.

**Results:**

Forty-nine patients underwent laparoscopic redo HM after failed primary HM. A new myotomy on the right lateral wall of the EGJ was the procedure of choice in the majority of patients (83.7%). In 36 patients (73.5%) an anti-reflux procedure was deemed necessary. Postoperative outcomes were somewhat less satisfactory, albeit comparable to the control group; the incidence of postoperative GERD was higher in the redo group (*p* < 0.01). At a median 5-year FU time, a good outcome was obtained in 71.4% of patients in the redo group; further 5 patients (10.2%) obtained a long-term symptom control after complementary PD, thus bringing the overall success rate to 81.6%. Stage IV disease at presentation was independently associated with a poor outcome of revisional LHD (*p* = 0.003).

**Conclusions:**

This study reports the largest case series of laparoscopic redo HM to date. The procedure, albeit difficult, is safe and effective in relieving symptoms in this group of patients with a highly refractory disease. The failure rate, albeit not significantly, and the post-operative reflux are higher than after primary HM. Patients with stage IV disease are at high risk of esophagectomy.

## Introduction

Laparoscopic Heller myotomy (LHM) has been considered in the last decades the gold standard of treatment for primary esophageal achalasia, providing symptom relief in 90% of the patients at a long-term follow-up.^1^,^2^ Despite these favorable results, about 10% of the patients will experience symptom recurrence (dysphagia, regurgitation, and chest pain) and will therefore require further treatment. Postoperative endoscopic pneumatic dilations (PD) are usually sufficient to provide symptom remission in most of these recurrencies.^2^ A minority of patients will however still experience persistent dysphagia and will therefore need revisional surgery, endoscopic myotomy,^3^ or even esophagectomy. Redo surgery after failed Heller myotomy (HM) nowadays still remains a controversial and poorly addressed topic. Most of the medical literature on the subject consists of retrospective case series including several different surgical procedures.^3^–^6^ The aim of our study was to assess the long-term outcome of revisional surgery after failed HM, the focus being on laparoscopic redo Heller myotomy.

## Materials and Methods

### Patients

All consecutive patients who were referred to our center for symptom recurrence after primary myotomy for achalasia and undergoing redo myotomy between September 2000 and December 2019 were enrolled in the study. Patients who underwent endoscopic myotomy or any surgical procedure on the upper GI **tract** between the primary operation and the redo procedure were excluded from the study. Patients’ demographics, clinical and surgical data, and postoperative follow-up were recorded prospectively in a dedicated database.

A control group was generated by matching patients who underwent redo myotomy (redo group) with those who underwent primary LHM at our center in the same time span (control group). Patients were matched for: age, sex, follow-up time, preoperative Eckardt score, previous endoscopic dilations, and radiological stage. For the purpose of randomization, a one-to-one nearest neighbor approach was used for the selection of patients in the control group. Postoperative outcomes were compared between the two groups, the primary outcome of interest was the failure of the surgical procedure, and the secondary outcomes were postoperative complications, length of hospital stay (LOS), and postoperative GERD. Lastly, subgroup univariate and multivariate analysis were conducted among patients who underwent redo surgery to identify potential risk factor for failure after redo myotomy.

### Preoperative Assessment

Patients symptoms were evaluated preoperatively using the Eckardt score.^7^ All patients had endoscopy to rule out other causes of dysphagia such as esophago-gastric malignancy or peptic strictures. Barium esophagogram was performed in all patients, and the degree of esophageal dilatation was quantified by measuring the maximum esophageal diameter at the barium-air interface. Patients were classified according to the diameter and shape of the lower esophagus as follows: grade I, 4 cm or less; grade II, 4–6 cm; grade III, 6 cm or more; and grade IV 6 cm or more and/or a sigmoid-shaped esophagus.^8^ Esophageal manometry was performed whenever accepted by the patients. Conventional (CM) or high-resolution (HRM) device was used according to the availability at the time of the evaluation. The protocols used for both techniques were extensively described in previous papers.^9^,^10^ Since all the subjects in the redo group already had an esophageal myotomy, the subgroup classification of achalasia according to the Chicago Classification was not considered.

### Causes of Failure of the Primary Procedure

Recurrences were definided as:

- *Early recurrences* (those occurring within 6 months after the primary procedure) included:
  - Incomplete myotomy, early scarring at the site of the myotomy or periesophageal fibrosis.
  - Obstruction due to a hyper-competent fundoplicatio.
- *Late recurrences* (those occurring after more than 6 months) included:
  c) Late scarring at the site of the myotomy, periesophageal fibrosis.
  d) Disease progression.
- In the presence of more possible causes, the factor that was perceived by the surgeon at operation as the most determinant was reported as the primary cause of treatment failure.

An incomplete myotomy was defined when a narrowing at the lower end of the myotomy at barium esophagogram and/or the persistence of a high pressure zone at the LES was identified^11^ and the length of the myotomy was confirmed to be inadequate intraoperatively. An obstructing fundoplication was defined as the persistence or early recurrence of dysphagia with the presence of a barium column narrowing or stasis at the EGJ, ab extrinseco compression at endoscopic retroversion of the cardia and the finding of an adequate myotomy length intraoperatively. The intra operative finding of scarring and periesophageal fibrosis was considered as the main cause of recurrence in the absence of other identifiable causes of symptoms recurrence.^6^ Finally, disease progression was only considered in late recurrencies and in the case of clear esophageal enlargement at consecutive barium esophagograms, in the absence of the other abovementioned causes of symptom recurrence.

### Surgical Technique for Redo Myotomy

All the procedures were approached laparoscopically. After division of all the adhesions, the first step of the procedure was to dismantle the fundoplication, when present. The esophagus was then circumferentially dissected and suspended with a Penrose drain. In this phase, if the esophagus appeared angulated or retracted because of adhesions, it was more extensively dissected from its mediastinal attachments and “straightened” in the abdomen for an adequate length. Once a “normal” anatomy was restored, a redo myotomy was performed, either by extending the pre-existing myotomy or by performing a new myotomy on the right side of the EGJ, more easily accessible. During this phase, a 30-mm Rigiflex balloon was placed at the cardia level using an endoscopically positioned guide wire and gently inflated to make residual circular muscle fibers more visible. When an anti-reflux valve was created, the Dor fundoplication was usually the procedure of choice; in some cases, however, where posterior detachment of the esophagus was particularly difficult and the previous Nissen fundoplication could not be safely completely dismantled, it was partially disassembled leaving its posterior portion in place and thus creating a Toupet-like fundoplication. Esophageal perforations identified intraoperatively were sutured by placing 4/0 interrupted absorbable stitches.

### Outcome Evaluation

Postoperative complications were graded according to the Clavien Dindo classification.^12^ Intraoperative complications and the conversions to open surgery were also recorded. The clinical outcome was assessed at 2, 6, and 12 months after surgery and every 2 years thereafter by administering again the preoperative symptom questionnaire. The follow-up protocol included a barium swallow at 2 months from the operation, esophageal manometry, and 24-h pH monitoring at 6 months and endoscopy at 1 year and every 2 years thereafter. Failure of the procedure was defined as the necessity for further surgical and endoscopic treatment or a postoperative Eckardt score higher than 3.^13^ Treatment failure after primary LHM in the control group was defined with the same criteria.

### Statistical Analysis

Data were expressed as medians and interquartile ranges (IQR) for continuous variables and as raw counts and percentages (%) for categorical variables. Continuous variables were compared using Mann-Whitney and Wilcoxon tests for unpaired and paired data, respectively. Categorical variables were compared using the Fisher’s exact test. Symptom-free survival estimates were calculated using the Kaplan-Meier method with log-rank tests for survival comparisons. All independent variables with associations of *p* = 0.1 at univariate analysis then underwent multivariate analysis: logistic regression models were used to identify independent predictors of redo myotomy failure. Odds ratios with 95% confidence intervals were calculated. A probability of 5% was assumed to be statistically significant (*p* = 0.05).

The study was approved by the Institutional Review Board of our Department. The patients signed an informed consent for the scientific use of their data.

## Results

During the same time frame, 1119 patients underwent primary Heller myotomy for Achalasia, and forty-nine patients underwent a revisional Heller myotomy with a laparoscopic approach after failed primary Heller myotomy. Eight of these patients (16.3%) had their first operation performed at our institution, and the remainder (83.7%) were referred after a first myotomy performed elsewhere. Patients’ demographics and clinical characteristics are summarized in Table 1. The majority of patients (75.5%) presented with recurrent dysphagia as the main complaint, whereas no patient referred with heartburn as the main presenting symptom. The median time between the first myotomy and symptom recurrence was 48 (2–72) months. An incomplete myotomy was identified at barium swallow or intraoperatively as the main determinant of failure of the first operation in the majority of cases (55.1%), presenting as a cause of both early and late recurrence. Details on the primary operations are reported in Table 1. Thirty-nine (79.6%) patients underwent a median of 2 (1–3) endoscopic dilatations between the first operation and the redo myotomy.

**Table 1:** Demographic and clinical characteristics of the studied population

N. of patients			49
Age (years)			41 (31–51)
Time between primary and redo procedure (years)			6 (3–13)
Median Eckardt’s score			7.5 (6–9)
**Main presenting symptom**		**N. of pts (%)**	
	Dysphagia		37 (75.5)
	Regurgitation		6 (12.2)
	Chest pain		6 (12.2)
**Cause and timing of symptoms recurrence**		**N. of pts (%)**	
Early recurrence			30 (61.2)
	Incomplete myotomy	19 (38.8)	
	Early fibrosis-scarring	4 (8.2)	
	Obstructing fundoplication	7 (14.3)	
Late recurrence			19 (38.8)
	Incomplete myotomy	8 (16.3)	
	Late fibrosis-scarring	6 (12.2)	
	Disease progression	5 (10.2)	
**Type of primary procedure**	**Type of associated fundoplication**	**N. of pts (%)**	
Laparoscopic myotomy			35 (71.4)
	Partial anterior fundoplication	26 (53.1)	
	Complete posterior fundoplication	7 (14.3)	
	Reconstruction of the His angle	1 (2)	
	No fundoplication	1 (2)	
Laparotomic myotomy			9 (18.4)
	Partial anterior fundoplication	4 (8.2)	
	Complete posterior fundoplication	4 (8.2)	
	No fundoplication	1 (2)	
Thoracotomic myotomy			5 (10.2)
	Partial posterior fundoplication	2 (4.1)	
	No fundoplication	3 (6)	
**Endoscopic dilations between primary and redo procedure**		**N. of pts (%)**	
Previous endoscopic dilations			39 (79.6)
	1–2 endoscopic dilations	18 (36.7)	
	> 3 endoscopic dilations	21 (42.9)	
No dilation			10 (20.4)

The redo myotomy was approached laparoscopically in all patients (Table 2); a new myotomy on the right aspect of the EGJ was the procedure of choice in the majority of cases (83.7%). In 36 patients (73.5%) the association of an anti-reflux procedure was deemed necessary, depending on the extension of the EGJ mobilization performed. The majority of them (77.8%) were fundoplications following Dor’s technique. Of the seven patients who underwent a Toupet fundoplication, 4 had a partial disassembling of a previous Nissen fundoplication. In 6 patients we chose to elongate towards the stomach a previous incomplete myotomy instead. Five of them had a fundoplication (Dor or Toupet), also.

**Table 2:** Surgical details of redo procedures

Type of redo procedure	Associated fundoplication	N. of pts (%)	
Laparoscopic new myotomy on the right aspect of the EGJ#			43 (87.8)
	No fundoplication	12 (24.5)	
	Dor fundoplication	25 (51)	
	Toupet fundoplication	5 (10.2)*	
	His angle reconstruction	1 (2)	
Laparoscopic prolongation of the previous myotomy			6 (12.2)
	No fundoplication	1 (2)	
	Dor fundoplication	3 (6.1)	
	Toupet fundoplication	2 (4.1)	

#Including one open conversion due to mucosal perforation

*In 4 of these patients a previous Nissen fundoplication was partially dismantled leaving the posterior portion in place thus transforming it into a Toupet-like fundoplication

Two intraoperative perforations occurred (4%) (Table 3): one was detected and sutured intraoperatively upon laparotomic conversion, and the second was detected at the upper GI series performed on POD 1: since the patient was symptomatic, we preferred open surgical revision to conservative treatment (Clavien Dindo grade 3b). Both patients were discharged on POD 13, without further complications.

**Table 3:** Analysis of postoperative results: redo pts and control group

	Redo patients (*n* = 49)	Control group (*n* = 49)	*p* value
**Matched variables**			
Sex (M/F)	22/27	27/22	0.42
Age	41 (31–51)	46 (34–53)	0.32
Follow-up time (months)	62 (24–151)	87 (44–125)	0.52
Eckardt score	2 (1–5)	2 (1–3)	0.20
Radiological stage			> 0.99
Stage I	8 (16.3%)	8 (16.3%)	
Stage II	23 (46.9%)	23 (46.9%)	
Stage III	10 (20.4%)	10 (20.4%)	
Stage IV	8 (16.3%)	8 (16.3%)	
Previous endoscopic dilations (yes/no)	39 (79.6%)	39 (79.6%)	> 0.99
**Results (unmatched outcome variables)**			
Operative time (min)	180 (144–222)	125 (110–150)	< 0.01
Intraoperative perforations (yes/no)	2 (4%)	1 (2%)	> 0.99
Hospital stay (days)	4 (3–6)	4 (3–6)	0.32
Postoperative complications (yes/no)	5 (10.2%)	0	0.06
Postoperative complications: Clavien Dindo			NA
Grade 1	1	0	
Grade 2	1	0	
Grade 3	3	0	
Grade 4	0	0	
Failure	9 (18.4%)	5 (10.2%)	0.39
Endoscopic Esophagitis	10/29 (34.5%)	0	< 0.0001
Abnormal 24 h pH study	11/26 (42.3%)	1/29 (3.5%)	0.0007
Overall GERD	16/29 (55.2%)	1/29 (3.5%)	< 0.0001

Two other patients required reoperation on POD 2 (Clavien Dindo grade 3b): both patients had evidence of complete obstruction at the upper GI series performed on POD 1 and were reoperated laparoscopically: the Dor fundoplication was dismantled and both patients were discharged without further complications on POD 4 and 7, respectively.

At a median follow-up of 62 (24–151) months, the outcome of the revisional surgery was favorable (Eckardt score < 3) in 71.4% of patients. Five additional patients (10.2%) achieved a long-term symptom remission after 2 complementary PD, thus bringing the overall success rate to 81.6%. The procedure failed in 9 cases (18.4%). Four patients did not improve after redo myotomy nor after complementary PD, still requiring periodic dilations to maintain acceptable symptom control or refusing further treatment. Two other patients underwent a second laparoscopic redo myotomy elsewhere. Esophagectomy was ultimately required in 3 patients (6%). All 3 of them had an early recurrence of dysphagia after the redo myotomy. All of them presented with a stage 4 disease.

**Fig. 1:**
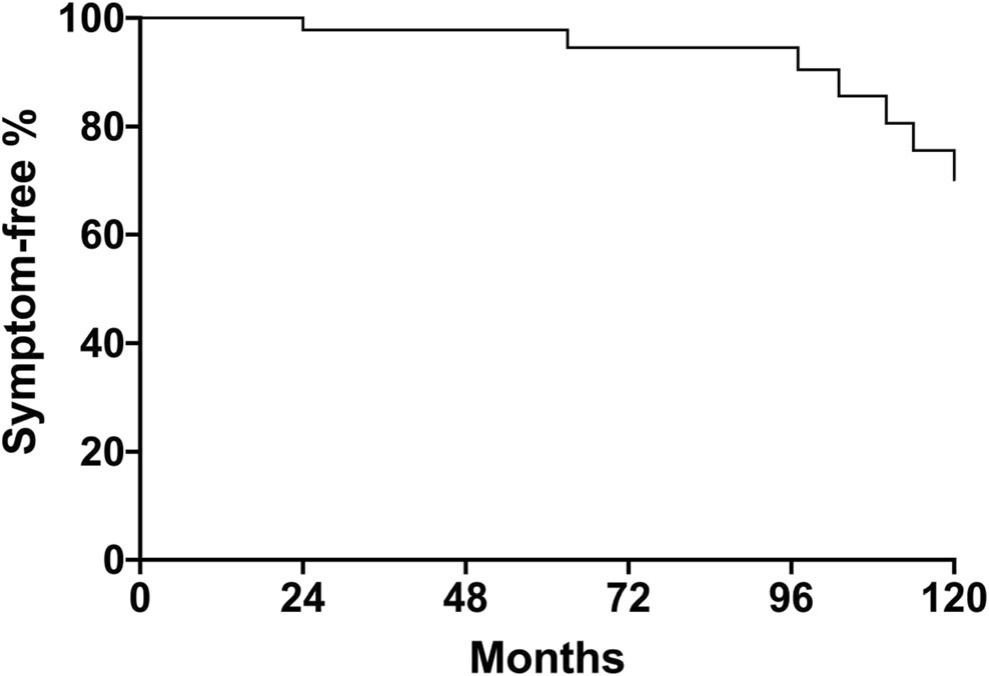
The Kaplan-Meier curve of symptom-free survival of the whole cohort of patients who underwent revisional myotomy.

At 5 and 10 years from surgery, the probability of being symptom free was 94.5 and 70.2%, respectively (Fig. 1).

Twenty-six patients (53.1%) agreed to undergo 24-h pH monitoring during the follow-up (Table 3). Eleven of these patients (42.3%) had a postoperative pathological acid exposure of the distal esophagus (Demeester >14.7). Data on postoperative endoscopy were available for 29 patients (59.2%). Esophagitis was present in 10 patients, 5 of them with a normal pH study, thus increasing the incidence of postoperative GERD to 55.2% (16/29).

**Fig. 2:**
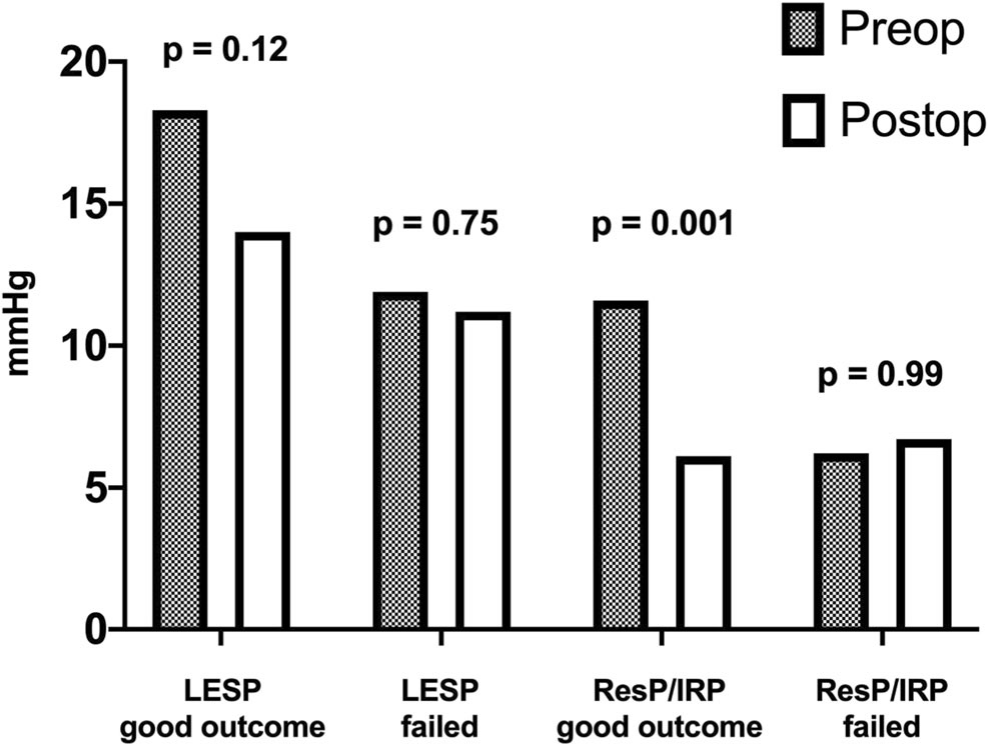
Manometric pressure parameters of the LES before and after redo myotomy. Preoperative (Preop) and postoperative (Postop) basal pressure (LESP) and relaxation pressure/integrated relaxation pressure (ResP/IRP) of patients with a positive (pos) and negative outcome (failed) after redo myotomy are reported. ResP/IRP was the only parameter showing a significant postoperative decrease only in patients who responded to redo myotomy (*p* < 0.01)

Preoperative and postoperative manometric data on the LES were available for 18 patients (36.7%), 15 with a favorable outcome after redo myotomy and 3 with symptoms recurrence. Data are summarized in Fig. 2. A significant decrease in the postoperative LES relaxation pressure (ResP/IRP) was detected among patients showing symptoms’ remission after redo myotomy (*p* = 0.001), while no decrease in such parameters was detected among patients who did not respond to treatment. Both preoperative LES basal pressure (LESP) and ResP/IRP were higher among patients with a positive outcome, compared with patients with treatment failure, even though these differences were not statistically significant (*p* = 0.32 and *p* = 0.41 respectively).

Compared to primary LHD performed at our Center in a matched cohort of patients (control group), redo procedures required a longer operative time (Table 3). The postoperative course was comparable between the two groups, but, although non-statistically significant, probably for the small number of cases, a trend towards a higher incidence of postoperative complications after redo myotomy was noted (10.2 vs 0%, *p* = 0.06). At follow-up, the failure rates of primary LHD and redo myotomy were not dissimilar (*p* = 0.39), even if, again, a trend towards a lesser favorable outcome was observed in redo patients. Finally, the incidence of postoperative GERD was significantly higher in the redo group (*p* < 0.01).

**Fig. 3:**
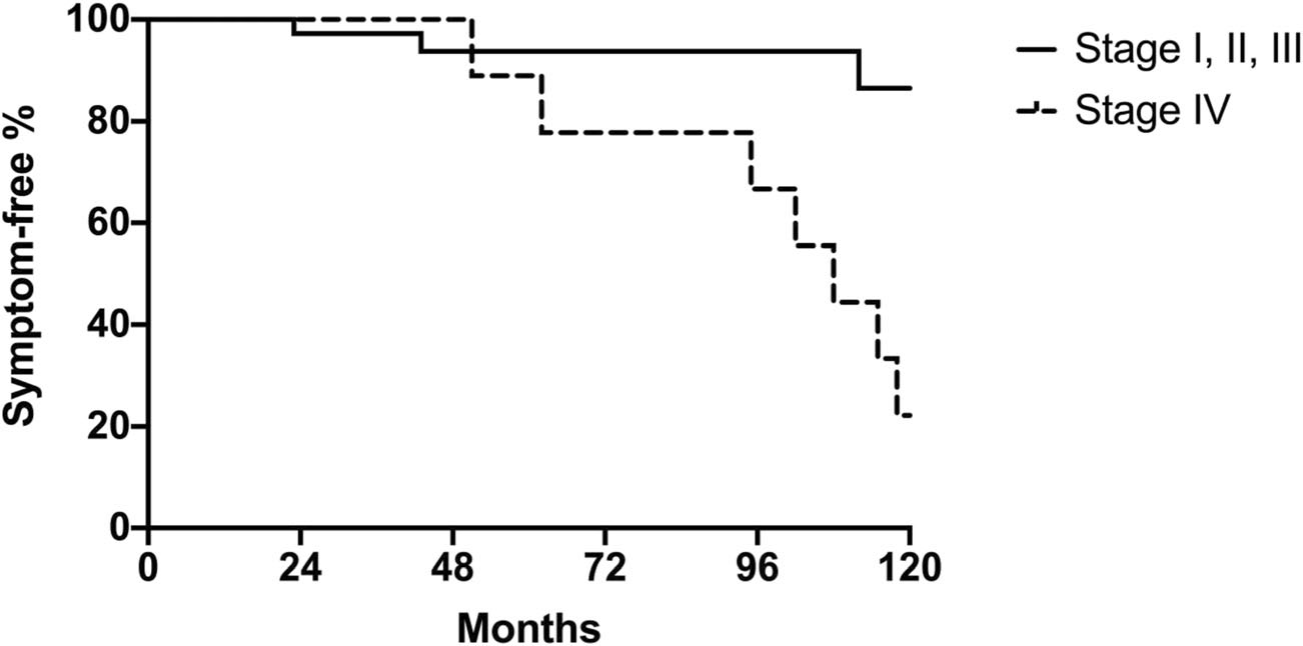
The Kaplan-Meier curve of symptom-free survival after revisional surgery by radiological stage. Patients presenting with stage IV disease had a significantly lower symptom-free survival after redo myotomy compared to patients with earler stages of the disease (*p* = 0.0002). These patients are probably responsible of the marked decline of Fig.1 curve, after 8 years of follow-up

At univariate analysis (Table 4), the presence of a preoperative symptom score > 5 (p = 0.03), the esophageal diameter (*p* = 0.04), and the presence of a sigmoid megaesophagus (stage IV disease) (*p* < 0.01) were all predictors of failure of the redo myotomy. The presence of a radiological stage IV disease was the only variable showing an independent association with a poor outcome on multivariate analysis (Table 5). Overall, 6 out of 8 patients (75%) with stage IV disease had symptom recurrence (see Table 3). The probability of maintaining symptom control 10 years after redo myotomy was 22.2% for patients with stage IV disease compared with 86.5% for patients presenting with earlier stages of disease (*p* < 0.01) (Fig. 3).

**Table 4:** Univariate analysis of risk factors for failure after redo procedures

Variables	Good outcome (*n* = 40)	Failure (*n* = 9)	*p* value
Sex (M/F)	19/21	3/9	0.49
Age	41 (31–50)	43 (37–51)	0.62
Time between first procedure and symptoms recurrence (months)	12 (1–72)	24 (2–30)	0.76
Early vs late symptoms recurrence			0.28
Early (30 patients)	26 (86.7%)	4 (13.3%)	
Late (19 patients)	14 (73.7%)	5 (26.3%)	
Eckardt score > 5 pre-redo (yes/no)	18/40 (45%)	8/9 (88.9%)	0.03
Esophageal diameter (mm)	46 (40-60)	63 (59–75)	0.04
Stage IV disease (sigmoid megaesophagus)	2/40 (5%)	6/9 (66.7%)	0.0001
Previous endoscopic treatment (39 patients)	31/40 (77.5%)	8/9 (88.9%)	0.66
Intraoperative mucosal perforation	2/40 (5%)	0/9	> 0.99

**Table 5:** Multivariate analysis of risk factors for failure after redo procedures

Binary variables	Odds ratio	95% CI	*p* value
Eckardt score > 5 pre-redo (yes/no)	3.35	0.27–86.45	0.37
Stage IV disease (sigmoid megaesophagus)	39.2	5.27–474.6	0.001

## Discussion

While there is general consensus in the literature regarding the role of laparoscopic HM as the gold standard treatment for esophageal achalasia,^2^,^14^ the approach of patients with symptom recurrence after myotomy remains a big challenge. Usually, a series of PD are recommended and offered to patients with recurrent symptoms, with satisfactory results in most of the patients.^2^,^11^,^14^ Sometimes, further treatment (i.e., a new surgical or endoscopic myotomy, or even esophagectomy) is necessary. The present paper represents, to the best of our knowledge, the largest case series published to date focusing on laparoscopic revisional myotomy.

The proportion of patients with a favorable outcome after redo myotomy was as high as 71.4% in our series, at a median FU of more than 5 years. This figure is comparable to those reported in other published series, where it ranged from 55 to 92%.^4^,^5^,^15^–^18^ However, the published literature on revisional surgery includes several different surgical procedures, frequently consisting of esophagectomies or simple dismantling of fundoplications rather than revisional myotomy. Moreover, the definition of failure is extremely variable or frequently not reported, thus making any comparison inconclusive.

The postoperative outcome of our cohort was comparable with that of a randomly selected group of matched patients, who underwent primary HD. This outcome should be cautiously interpreted, given the relatively small number of patients and the inevitable loss of data due to the selection of the control group. However, the figures are also comparable to those reported by our group after primary HD in a recently published series of more than 1000 patients.^2^

When planning a revisional surgery, a complete resolution of symptoms may hardly be expected, and the main goal of the operation should rather be an improvement in patients’ quality of life and the maintenance of their oral feeding capacity,^5^ without converting them to esophagectomy. A thorough preoperative evaluation is therefore mandatory to correctly identify those who would most likely benefit from a revisional operation. Barium esophagogram probably represents the most useful tool in the evaluation of patients with recurrent dysphagia after myotomy. Firstly it provides valuable information about the degree of esophageal dilation and the residual clearing capacity of the organ, and secondly it helps determining the undermining cause of recurrent dysphagia.^19^ Timed esophagogram may be the best way to evaluate the patients,^20^ but it is not widespread among radiologists, therefore not always available. Loviscek et al.^4^ also underlined the role of barium esophagogram as a prognostic tool. The authors report a 75% success rate after revisional surgery for radiological stage I and II that compared favorably with a 33% rate of symptoms remission for stage IV patients. Our series showed comparable results by using a similar radiological staging,^8^ with a 75% failure rate among patients with stage IV disease.

Only a few of the redo myotomy series report the manometric data pre- and post- re-treatment. Gockel et al.^16^ described a significant reduction in LES resting pressure after re-myotomy in patients with early symptom recurrence; however no control manometric data on patients with failure of re-treatment was provided. In the present series, the LES relaxation pressure (ResP/IRP) was the only parameter demonstrating a potential correlation with the outcome of redo myotomy, showing a significant decrease only in patients who responded to re-treatment. Interestingly, even though no significant difference was detected, patients with a good outcome after redo myotomy showed higher preoperative LES basal and relaxation pressures compared with failed patients. The redo operation reduced these values to a level of the preoperative ones of the patients who did not benefit from the second operation that remained unchanged (Fig. 2). In light of these findings, it can be hypothesized that the defective body motility or other functional alterations could be the main cause of treatment failure among these subjects. Despite the limitations due to the paucity of patients with available data in this study, we concluded that esophageal manometry is not a good prognostic tool for patients who are candidate for redo-myotomy, nor should be used to guide treatment decisions.

The surgical technique routinely adopted at our center is standardized and similar to that proposed by other authors.^4^,^5^,^19^ Some authors advocate prolonging the previous myotomy when this is clearly identifiable, reserving the creation of a new myotomy on the lateral side of the esophagus only when an extensive fibrotic reaction on the site of the previous one is evident.^4^,^5^ Others favor the systematic use of a new myotomy on the right lateral aspect of the EGJ.^19^ Our preference goes to the latter approach for several reasons. It is usually difficult to identify the previous myotomy, and to conduct a new dissection on a scarring area harbors a higher risk of perforation. As previously described for primary HD,^2^ we usually extend the new myotomy for 7–8 cm on the esophageal side and for 2 cm minimum on the gastric side.

As previously reported by our group for primary Heller myotomy patients,^21^ we sought to perform postoperative pH monitoring after revisional surgery, whenever tolerated. We believe this is especially important in this complex subset of patients who underwent many surgical and endoscopic procedures and for whom is, therefore, always difficult to discern the etiology of postoperative symptoms (particularly chest pain, heartburn, and regurgitation).

While performing a fundoplication significantly reduces the incidence of postoperative reflux after primary myotomy,^22^,^23^ there is no comparable evidence in the literature regarding redo surgery. Both Veenstra et al.^5^ and Loviscek et al.^4^ report performing a fundoplication only in a minority of patients undergoing revisional procedures, to minimize the risk of postoperative dysphagia. Both authors do not report the data on postoperative reflux. The incidence of postoperative GERD after redo myotomy reaches 55.2% in our series, resulting significantly higher than the control group (3.5%, *p* < 0.01) and being significantly higher than what our group and the majority of papers reported after primary HM.^2^ Giving these results, it may be questionable to still perform a fundoplication. We still try, however, to always perform a new fundoplication unless contraindicated by an excessive risk of lesions or postoperative persistent dysphagia. While other authors^4^,^19^ report the use of a Toupet fundoplication, reserving a Dor only for cases with suspected intraoperative perforation, our preference goes to the latter, since it allows to neatly cover the new myotomy site, especially when an extensive posterior mobilization of the EGJ was not necessary.

In the last decade, Per-oral endoscopic myotomy (POEM) had a widespread diffusion, clearly challenging the laparoscopic myotomy as the treatment of choice for primary achalasia. Definitely, POEM may play an important role in treating symptoms of achalasia recurring after laparoscopic myotomy also. The use of POEM in this context was first reported by Zhou et al. in 2013^24^ in a prospective study on 12 patients, with a success rate of 91.7%. The largest case series on the topic are reported in two multicentric studies: Ngamruengphong et al.^25^ conducted a retrospective cohort study on 90 patients treated with POEM for achalasia who had previously undergone a Heller myotomy. The symptoms’ remission rate was 81%, with a 4% incidence of mucosal tear and 1 patient requiring thoracoscopic drainage of the mediastinum for leakage. Tyberg et al.^26^ retrospectively evaluated 51 patients referring from 13 centers who underwent POEM for symptoms’ recurrence after myotomy, the success rate in the series was 94%, 6 patients experienced intraoperative perforation treated endoscopically, and 2 developed mediastinitis treated conservatively. The use of POEM allows surgeons to revise the myotomy site without facing the challenges of a trans-abdominal procedure in this complex setting. Given the encouraging results obtained in these studies, POEM surely represents a valuable alternative to surgery, especially considering the high frequency of postoperative GERD after revisional surgery, similar or even superior to that usually reported after POEM. However, complications after POEM, albeit rare, are not nihil and may be serious. Some points remain opened for discussion: first, the follow-up time of the reported studies ranges between 2 and 24 months; therefore further evaluation needs to be achieved on the long-term results of this technique. Second, while POEM would surely represent a valuable option in patients with an incomplete myotomy as the sole cause of recurrent dysphagia, the relapse of symptoms after failed myotomy could be more frequently the result of several overlapping phenomena, including tissue scarring, periesophageal fibrosis, or an obstructing fundoplication. In these cases, it is not clear whether a POEM would suffice or a laparoscopic procedure would be more likely to relieve the symptoms.^6^ Of course, more studies are needed to answer this and other questions, even if comparative studies among different procedures are unlikely, due to the rarity of esophageal achalasia and the even rarer failures of available treatments.

Finally, several authors recommend esophagectomy for the treatment of end-stage achalasia or recurrent dysphagia after Heller myotomy. Orringer et al.^27^ reported the restoring of a normal alimentation in 96% of patients, and similar results were described by Devaney et al..^28^ In a recent propensity matched analysis, Molena et al.^29^ evaluated the post-surgical outcome concluding that esophagectomy for end-stage achalasia and for cancer offer comparable results in terms of post-operative morbidity and mortality. However, the study did not take into account several procedure-specific complications (anastomotic leak, vocal cord palsy, chyle leak) with a remarkable impact on the postoperative course. In our series, the presence of a stage IV disease after primary HM represented an independent risk factor for failure of the redo myotomy. It could be therefore argued that these patients should have been managed with resection rather than an esophageal-sparing procedure. Esophagectomy is, however, burdened by a rate of postoperative complications ranging from 20 to 80% and an in-hospital mortality reaching 20%.^30^,^31^ Furthermore several factors make esophagectomy for achalasia more technically demanding than esophagectomy for cancer, including the displacement of mediastinal structures due to esophageal dilation, the increased risk of bleeding due to the hypertrophy of the muscular layer, and the scarring due to the previous procedures.^28^ Our results confirmed that redo myotomy is a safer procedure in terms of postoperative outcome. While LHD is effective in 75% of the subjects with stage IV primary achalasia,^2^ in this study a new myotomy was effective in 25% of redo patients presenting with advanced disease only. However, since this approach may spare patients the burden of a more invasive operation, we believe that redo myotomy, in experienced hands, should still be offered as the first option for patients presenting with recurrent symptoms and a decompensated megaesophagus also. The patients, however, should be made very well aware of the risk that this new myotomy could be effective in a minority of cases only and the risk of a new, bigger operation is always present.

Notably, the majority of patients were referred after a first failed procedure performed elsewhere (83.7%). In a previous paper of ours, we already discussed the importance of case-volume in determining surgical outcomes after primary Heller Myotomy.^2^ Between 16 and 20 procedures are considered necessary to complete the learning curve, and this is not readily achievable in such a rare disease as achalasia.^32^,^33^ Analysis of large national-based registries have already demonstrated how the case-volume of Heller myotomy influences the postoperative outcome.^34^ Taking this into consideration, our series of patients may express the sub-optimal expertise of some centers and prompt the need for centralization of such complex cases.

## Conclusion

Redo laparoscopic myotomy is a feasible, albeit complex, surgical procedure, that proved to be effective in the treatment of an extremely complex subset of patients with recurrent or persistent dysphagia after primary myotomy. When performed in experienced centers, the success rate, regardless the cause of symptom recurrence, is comparable to that obtained after primary myotomy for achalasia, provided that a thorough preoperative evaluation is performed and an adequate standardization of the operation is adopted. Patients presenting with megaesophagus are at higher risk of treatment failure even after redo myotomy.
